# Low-sample supervised fault diagnosis for fixed-wing UAVs based on multi-scale adaptive state-aware sequence learning

**DOI:** 10.3389/fpls.2026.1805999

**Published:** 2026-04-14

**Authors:** Min Li, Long xia Zhu, Jing Yan, Jia tong Zhang, Hai feng Fan, Su juan Liu, Ming Gao

**Affiliations:** 1 College of Artificial Intelligence, Tianjin University of Science and Technology, Tianjin, China; 2 Tianjin Yunsheng Intelligent Technology Co., Ltd., Tianjin, China

**Keywords:** adaptive state selection mechanism, deep learning, fault diagnosis, smart plant protection, unmanned aerial vehicles

## Abstract

With the rapid development of smart agriculture and plant protection applications, unmanned aerial vehicles (UAVs) systems that integrate various advanced technologies have become increasingly important for enhancing agricultural production efficiency and reducing operational costs. In order to ensure the safe and stable operation of UAVs, fault diagnosis and reliability evaluation research are very important. Existing fault detection methods often have the problems of unsatisfactory accuracy in low-sample conditions, low computing efficiency and insufficient extraction of temporal characteristics, which limits their reliability and practical applicability. The challenge is further compounded by the scarcity of labeled fault data in real-world applications, underscoring the critical need for low-sample learning methods. To address these issues, this research proposes a multi-scale adaptive state-aware sequence learning framework. The framework adopts a Mamba-enhanced multi-scale temporal network (Mamba-MSTN), which integrates the Multi-Scale Temporal Feature Extraction (MSTFE) module, the adaptive state perception and screening module based on the selection state space model Mamba, and the global dependency modeling module based on Multi-Head Self-Attention (MHSA). Specifically, the MSTFE module hybridizes a 1D Residual Convolutional Neural Network (1D-RCNN) with Bidirectional Gated Recurrent Units (BiGRU) for jointly capturing transient details and long-term trends in flight data, thereby enabling effective multi-granularity temporal feature extraction. Mamba is introduced as an adaptive state perception and screening module. The module adopts an input-dependent state conversion mechanism to dynamically model flight timeseries data in a content-aware way, so as to realize key information filtering. The MHSA mechanism enhances the global dependency representation through parallel multi-subspace modeling adaptive attention to key segments, thus making up for the limitations of local modeling. Comprehensive experiments prove that the proposed method shows high sensitivity and robust generalization in binary and multi-class low-sample fault diagnosis tasks. It is superior to the current mainstream method in terms of accuracy, processing efficiency and resource consumption, and has strong practical application potential.

## 1 Introduction

Plant protection is the fundamental task of ensuring the safety of agricultural production, maintaining a stable food supply, and supporting the sustainable development of agriculture. Crops are vulnerable to biological stresses such as diseases, pests, and weeds during growth, resulting in declines in yield and quality. This situation threatens food security, reduces farmers income, and has a chain effect on the food safety system, thus seriously affecting the socio-economic landscape (Chin et al., 2023). Plant protection is a key means of achieving stable growth in crop yields. If diseases are not addressed at an early stage, the difficulty and cost of control will increase significantly (Abbas et al., 2023). Plant protection also promotes the green and sustainable development of agriculture. Although excessive or indiscriminate use of pesticides can temporarily suppress pests and diseases, it leads to environmental pollution, pesticide residues, and increased pest resistance, thereby threatening the long-term stability of agricultural ecosystems (Guebsi et al., 2024). Traditional plant protection methods rely on manual field inspection or empirical judgment, which are not only inefficient but also unable to cover large agricultural areas or detect disease symptoms at an early stage. In recent years, with the development of precision agriculture and smart agriculture, plant protection has evolved from a manual model into an advanced system integrating remote sensing, big data, and intelligent decision-making (Guebsi et al., 2024; Hafeez et al., 2023). Data-driven plant protection systems enable early intervention and precise control of crops, effectively suppressing disease spread, reducing pesticide application intensity, and minimizing yield losses and ecological risks.

In the field of smart plant protection, obtaining efficient and reliable field information is the key to achieving precise intervention. With its flexibility and wide coverage, UAVs technology can quickly collect high-quality crop information, which plays an increasingly important role in modern precision agricultural systems. Specifically, in plant disease monitoring, UAVs equipped with visible light, thermal infrared and other sensors can realize fine perception of crop growth status and disease characteristics (Chin et al., 2023; Abbas et al., 2023). For example, UAVs remote sensing can identify subtle changes in crop leaf color, texture and spectral characteristics that are difficult to observe with the naked eye, thus providing a basis for supporting disease intervention (Chin et al., 2023; Shafik, 2024). The accuracy and efficiency of illness detection can be greatly increased by integrating sophisticated algorithms like deep learning, which can automatically analyze and process the data images gathered by UAVs (Pajany et al., 2024). In the actual plant protection application, UAVs can accurately locate the ward, realize variable-speed spraying and accurate application, avoid excessive spraying, and reduce environmental pollution (Wang et al., 2023). Chen and Lu (2025), proposed the KAN-Former model to realize high-precision 4D trajectory prediction by modeling the physical correlation and long-term timing-dependent characteristics of flight variables, which can provide a more stable state estimation for complex operations; Yan et al. (2024) improves the ant colony optimization algorithm and designs an adaptive obstacle avoidance strategy to realize the efficient generation of the optimal trajectory of the drone under the comprehensive target function constraints, which can dynamically optimize the path and reduce the trajectory loss rate in an uncertain obstacle environment. The above trajectory optimization and prediction algorithms can improve the continuity and stability of the flight path. It can be applied to plant protection UAVs, which can enhance the system’s adaptability to uncertain factors such as complex farmland terrain and sudden meteorological disturbances, reduce the phenomenon of leakage and re-spraying, and improve the uniformity of drug application and operation safety. However, in terms of production costs and operational efficiency, traditional manual plant protection methods are facing increasing sustainability challenges due to rural labor shortages and rising labor costs. The automation and intelligence of UAVs provide solutions to support large-scale agricultural production (Hafeez et al., 2023). In addition, UAVs maintain strong operating capabilities in complex terrain and high-density crop environments, further improving the efficiency of plant protection operations.

In practical plant protection operations, UAVs usually operate at low altitude for a long time in bad weather conditions and complex agricultural environments. These operation requirements accelerate mechanical wear and sensor aging, thus reducing the safety and effectiveness of plant protection tasks (Toscano et al., 2024). UAVs may encounter sensor or control system failures, which disrupt data collection, lead to uneven pesticide application, and even lead to crashes and secondary losses. Agricultural spraying UAVs rely on the coordinated operation of key components such as brushless DC motors, electronic speed regulators, pumps, nozzles and GPS navigation aircraft systems. The failure of any of these components may affect the overall spraying accuracy. For example, motor failure or electronic speed regulator signal deviation may lead to unstable flight posture (route offset), resulting in uneven distribution of pesticide spraying, including local overspraying or undercoverage. With the help of fault diagnosis technology, it can actively identify potential faults during the flight, and dynamically adjust the pump speed, nozzle flow and flight parameters in a timely manner, which can solve this defect. For example, the plant protection UAVs variable spraying system based on Artificial Neural Network realizes the precise adjustment of the mist droplet deposition rate, so that the spraying error rate is controlled within 20% (Wen et al., 2019). In addition, crop health detection UAVs rely on their special sensors to capture high-resolution and high-precision image data. Real-time monitoring platforms and deep learning models (such as Convolutional Neural Networks (CNN) used for disease identification) based on the Internet of Things (IoT) are relatively sensitive to the quality of input data, and their monitoring accuracy can reach 99.53% (Ferentinos, 2018). However, this premise is that the working state of the sensor is stable. Once the sensor fails, it will cause data distortion or omission. For example, the failure of the thermal imaging camera may cause inaccurate crown temperature measurement, thus misjudging the level of crop moisture stress. By introducing improved fault diagnosis methods, the sensor status can be verified in time and reliable support can be provided for accurate agricultural protection decision-making. Continuously collect multi-modal data during plant protection operations to realize online health monitoring and early warning of failures, ensure the stable implementation of tasks, reduce the risk of crashes, and protect personnel and crops. Precise fault diagnosis helps to identify faulty components and their root causes, thus avoiding unnecessary returns to the manufacturer and blind replacement of components, thus reducing operating costs and system downtime. As the complexity of current UAV systems continues to increase, their operational status depends on multiple factors, making post-failure maintenance alone insufficient to meet the requirements of high-frequency and high-intensity plant protection operations (Toscano et al., 2024). Existing machine learning and deep learning methods demonstrate strong capabilities in anomaly detection and fault identification. These diagnostic models can rapidly detect sensor or data anomalies to prevent low-quality or erroneous data from entering disease recognition model training and online inference, thereby improving the accuracy and reliability of plant protection decision-making. For instance, analyzing the continuous collection of multisource data, such as flight control parameters and sensor outputs, allows real-time system health monitoring and anomaly identification (Toscano et al., 2024). In scenarios involving multiple UAVs or UAV swarms, fault diagnostics also provide guidance for task reallocation and fault-tolerant control to enhance overall system robustness and increase task completion success rates (Ali et al., 2024).

Currently, the existing UAV fault diagnosis methods mainly follow three technical paths (Li et al., 2022): hardware-redundancy-based methods, analytical model-based methods, and data-driven methods. Hardware redundancy achieves fault tolerance by adding physical backups of key components, so that multiple identical or similar devices can run in parallel. This method is known for its high reliability and has been widely used in the aerospace industry (Najjar et al., 2016; Deckert et al., 1977). However, due to the limitations of the size, payload capacity, and manufacturing cost of the UAV platform, the large-scale implementation of hardware redundancy is still challenging for UAVs.

The analytical model-based methods entail creating a precise mathematical or physical model of the UAV to build observers or filters for fault diagnosis. The residuals between the model-predicted value and the actual measurements can function as effective indicators of system faults (Gong et al., 2023). Specifically, Zuo et al. (2020) proposed combining an unknown input observer with a feedback compensation fault-tolerant controller to address fault-tolerant control and fault diagnosis of quadrotor UAVs under nonlinear dynamics, external interference, and inertial measurement unit sensor faults. To address the actuator fault detection and diagnosis problem in tilt-rotor UAVs under non-redundant actuator conditions, Gao et al. (2020) used a combination of extended Kalman filter and multiple model adaptive estimation to solve the actuator fault detection and diagnosis problem of tilt-rotor UAVs under non-redundant actuator conditions. Although the model-based method is theoretically systematic and can achieve effective diagnosis in specific scenarios, its performance largely depends on the accuracy of the mathematical model of the controlled system and the accumulation of expert knowledge. UAVs exhibit complex aerodynamic characteristics and dynamic coupling among multiple variables during flight, making it difficult for theoretical models to accurately capture their actual motion behavior. The resulting model mismatch often leads to false alarms or missed detections in diagnostic systems. In addition, acquiring expertise in specific domains itself poses a major challenge, further limiting the practical application of this method in UAV fault diagnosis.

The data-driven method represented by deep learning can significantly reduce the dependence on precise models and reduce the cost of modeling. In addition, well-trained diagnostic models can be transferred to different platforms or task scenarios, showing better versatility. These methods do not require prior knowledge and can automatically extract fault characteristics from data, showing obvious advantages, and gradually becoming research a research focus in this field (Kang et al., 2022; Liu et al., 2022). Algorithms for deep learning such as Long Short-Term Memory (LSTM) networks (Wang et al., 2020; Guo et al., 2023), CNN (Guo et al., 2018; Ma et al., 2024), Gated Recurrent Units (GRU) (Masalimov et al., 2022), and Deep Forest (Yao et al., 2023) have achieved substantial advances in UAVs fault diagnosis. For instance, Guo et al. (2018) introduced a hybrid feature model based on CNN and short-time Fourier transform, whereas Du et al. (2022) utilized interval sampling and reconstruction of vibration signals with a 1D-CNN. For different types of fault scenarios in UAVs, targeted anomaly and handling of anomalies and faults are achieved. Park et al. (2022) proposed an unsupervised method for anomaly detection, which does not rely on system models or fault labels. Liu et al. (2023) designed an ensemble transfer learning method using BiLSTM. Yang et al. (2023) suggested an unsupervised approach using spatiotemporal correlation using an LSTM and autoencoder, combining Savitzky-Golay filtering for noise reduction and correlation feature selection. In order to combine the advantages of CNN and LSTM, Huang et al. (2024) introduced a weighted ensemble method, comprising CNN, BiLSTM, and BiGRU. Kumar et al. (2024) proposed a low-power, high-efficiency fault detection method integrating lightweight CNN, LSTM, and attention mechanisms. Zhang et al. (2024) proposed a heterogeneous deep multi-task learning framework based on adaptive sharing and knowledge complementarity. To handle the complex, high-dimensional, and multi-source heterogeneous spatiotemporal data in UAVs systems, Transformer-based models (Peng et al., 2024; Li et al., 2025; Zhang et al., 2025) and their variants (e.g., Informer (Ma et al., 2025)) exhibit strong capabilities in feature extraction and fault identification. These models significantly enhance diagnostic accuracy and robustness in challenging scenarios, such as low-sample learning, cross-domain tasks, and swarm coordination. For example, Peng et al. (2024) designed multiscale spatial temporal Bayesian graph conv-transformer approach, which mines spatial correlations among UAVs swarms via graph attention networks, captures multi-scale temporal features with convolutional Transformers, and quantifies diagnostic uncertainty using Bayesian deep learning, achieving highly reliable distributed fault diagnosis for fixed-wing unmanned aerial vehicles (FW-UAVs) swarm systems. Li et al. (2025) designed multi-scale fault detection technique, integrating Gated Graph Convolutional Networks (GatedGCN) and CNN-Transformer. By jointly modeling the spatial topological relationships among sensors and temporal dynamic patterns, it effectively addresses the issue of fault feature confusion in fixed-wing UAVs caused by closed-loop control, enhancing flight state cognition and fault identification accuracy. Zhang et al. (2025) suggested multi-modal deep learning, using Transformer-TCN and recursive feature fusion map, effectively integrating six types of time-series signals such as position, velocity, and current to achieve high-precision diagnosis and health assessment for actuators in flying-wing UAVs under both single and compound fault modes. Ma et al. (2025) proposed a multi-head attention architecture integrating 1DCNN and Informer. Signal preprocessing is performed through a dual-path parallel process, where variational mode decomposition extracts long-range dependencies and the Fast Fourier Transform (FFT) captures local temporal features. This allows for high-precision autonomous diagnosis of motor bearing problems in UAVs. Hu et al. (2025) designed multi-scale Transformers with comparative learning. This method adaptively selects the temporal scale through the expert hybrid architecture and uses the dual-attention mechanism for temporal correlations and local dependencies within the flight data. Modeling effectively solves the abnormal detection challenges caused by time changes and complex correlations in UAVs flight data. The current deep learning-based fault diagnosis techniques either have a high computational load and low processing efficiency, or they struggle to completely capture multi-scale dependencies in temporal data, which leads to performance and resource consumption flaws.

In recent years, the State Space Models (SSMs) has become an important example of sequence modeling. Due to its linear time complexity and strong ability to capture remote dependencies, it has demonstrated the potential to replace traditional attention mechanisms in various fields (Zhang et al., 2023) (Nguyen et al., 2023). However, the classic linear time-invariant SSMs is limited by the static conversion mechanism, which limits its ability to dynamically adjust the information retention strategy according to the input content. When dealing with complex time patterns that require context-sensitive reasoning, it is usually less expressive than attention-based architectures. In order to overcome this limitation, Gu and Dao (2024) proposed Mamba, a selective SSMs, introducing state transformation parameters that depend on input to achieve dynamic screening of sequential information and content perception modeling. Compared to the Transformer, Mamba achieves nearly linear complexity, enhancing both efficiency and performance in long-sequence tasks. This progress has quickly stimulated the application of Mamba in the field of fault diagnosis. For example, Wang et al. (2025a) proposed a fault diagnosis method called MD-BiMamba, which combines complete integrated empirical mode decomposition with adaptive noise signal decomposition with bidirectional Mamba network. This method effectively solves the challenge of achieving high-precision, low-complexity fault diagnosis of intermediate bearing sensor signals in aircraft engines, even amidst high-dimensional complex noise interference. Shen et al. (2024) proposed a bidirectional Mamba predictive battery temperature representation architecture for battery temperature prediction and fault diagnosis. By combining a two-stage hybrid data preprocessing approach and a multi-step prediction mechanism, it effectively resolves difficulties in temperature modeling and delayed thermal runaway warnings for electric vehicle batteries under conditions of noise interference, dynamic operating conditions, and data scarcity. Wang et al. (2025b) suggested Mamba model integrated with feature mode decomposition and selection, achieving high-precision diagnosis of weak faults across operating conditions with strong noise resistance, demonstrating superior performance on engine and induction motor datasets. Xie et al. (2024) used Mamba and its two-way variant BiMamba as a SignalMixer component to build a lightweight fault diagnosis model MetaNet, verifying the future of Mamba as a mechanical fault diagnosis of gearbox data sets. Although Mamba has shown excellent performance in the above-mentioned fault detection tasks, there has been no substantial exploration in the key areas of fault diagnosis of FW-UAVs flight systems.

Against this background, the existing fault diagnosis methods face three bottlenecks. First, model’s capacity to extract and incorporate intricate temporal features restricts the potential for enhancing diagnostic accuracy. Second, the mainstream deep learning architecture usually requires numerous parameters and high computing load, resulting in obvious delays in training and reasoning, which ultimately hinders the actual engineering. Third, existing methods suffer from suboptimal diagnostic accuracy under low-sample conditions and insufficient temporal feature extraction, making it difficult to simultaneously capture transient details and long-term dependencies in flight data. The proposed method overcomes these bottlenecks at the same time and meets the application requirements of accuracy, feature representation ability and efficiency. The limited availability of fault data in practice has further exacerbated this problem, making low-sample learning an indispensable requirement for real-world deployment. The principled solutions of these bottlenecks require the collaborative integration of multigranular feature learning and efficient state space modeling. 1D-RCNN and BiGRU capture transient details and long-term dependence respectively. Then, Mamba performs adaptive context-aware filtering with linear complexity to directly reduce the computational burden while retaining diagnostic-related information. Finally, MHSA has established global interdependence to make up for the limitations of local modeling. This paper proposes the Mamba-MSTN framework. The framework integrates the multiscale time feature extraction (MSTFE) module, the dynamic state perception and screening module based on Mamba, and the global dependence modeling module based on multi-head self-attention (MHSA). This closely integrated architecture solves both accuracy limitations and efficiency requirements.

The main contributions of this paper can be summarized as follows:

This study introduces Mamba into the field of FW-UAVs fault detection for the first time. As an adaptive state perception and filtering component, Mamba’s selection mechanism can dynamically track and filter key information in flight timing data, and achieve efficient sequence modeling with its linear time computing efficiency.

The MSTFE module can capture short-term fluctuations and long-term laws in flight data in real time, so as to extract the timing characteristics of different scales. At the same time, the module combines the MHSA mechanism. Through parallel multi-subspace modeling, it adapts attention to key parts related to faults, improves the model’s understanding of the overall correlation of data, and alleviates the defects caused by focusing only on local patterns.

The experimental results on the real FW-UAVs fault datasets (Bronz et al., 2020) show that the proposed Mamba-MSTN framework has achieved a coordinated improvement of diagnostic accuracy and computing efficiency in binary-classification and multi-classification fault detection tasks, and the comprehensive performance reaches leading level.

This article is structured as follows: Section 2 covers the necessary background, presents the design of the Mamba-MSTN model, and describes the experimental setup. Section 3 evaluates the performance of the proposed method through a detailed analysis of the results. Finally, Section 4 discusses the implications of the study and outlines potential future work.

## 2 Materials and methods

### 2.1 Relevant technical knowledge

#### 2.1.1 Temporal modeling mechanisms

1. SSMs and Mamba: The main advantage of SSMs is that it can model long-range dependencies with a computational complexity that is linear or almost linear with the sequence length, and provides a rigorous mathematical framework rooted in control theory. SSMs is a sequence model whose core is composed of a set of linear differential equations, which use the potential state $h\left(t\right)\in {\mathbb{R}}^{N}$ to transfer input sequence $x\left(t\right)\in {\mathbb{R}}^{N}$ to output sequence $y\left(t\right)\in {\mathbb{R}}^{N}$, as in the equation (1):

(1) $$h^{\prime }\left(t\right)=\text{A}h\left(t\right)+\text{B}x\left(t\right)y\left(t\right)=\text{C}h\left(t\right)$$

where state matrix is denoted by $\text{A}\in {\mathbb{R}}^{N\times N}$, and projection matrix are denoted by $\text{B}\in {\mathbb{R}}^{N\times 1}$ and $\text{C}\in {\mathbb{R}}^{1\times N}$.

Discrete SSMs (Zhang et al., 2023; Gu et al., 2020) use a parameter called Δ to turn the system above into a discrete form as follows shown in the equation (2):

(2) $$h_{t}=\bar{A}h_{t-1}+\bar{B}x_{t}\;y_{t}=\text{C}h_{t}$$

where *x_t_*, *y_t_* and *ℎ_t_* are the input, output, and state vectors at time t. $\bar{A}$ and $\bar{B}$ are obtained by using the following discretization rule, as shown in the equation (3).

(3) $$\bar{A}=\text{exp}\;(\Delta \text{A})\bar{\text{B}}=\left(\Delta \text{A}{)}^{-1}\left(\text{exp}\;(\Delta \text{A}\right)-\text{I}\right)\cdot \Delta \text{B}$$

Here, the parameter used in the discretization rule is called Δ.

As shown in earlier work by Gu et al (Gu et al., 2020), this discrete model can actually be written as a convolution. It looks like shown in the equation (4):

(4) $$\bar{K}=\left(\bar{CB},\bar{CAB},\cdots ,{\bar{CA}}^{M-1}\bar{B}\right)\;y=x*\bar{K}$$

Here, M is the length of the input sequence. Discrete SSMs have a structure that looks a lot like a standard Recurrent Neural Networks (RNNs) (Elman, 1990). But there’s a key difference: Discrete SSMs can be computed in parallel much more efficiently traditional RNNs can’t easily do, since they depend on nonlinear activation functions that force them to process the sequence step by step.

In traditional SSMs, the system matrix A, B, C and the discrete step length Δ are usually set to constant parameters unrelated to temporal input. While this time-variant characteristic facilitates system analysis, it simultaneously restricts the model’s ability to dynamically adjust its state transitions and output responses in accordance with the input context. Consequently, this limitation impairs the model’s effectiveness in complex sequence modeling (Qu et al., 2024). In order to overcome this limitation (Gu and Dao, 2024), introduced a novel sequence modeling architecture called Mamba. Modeling the SSM parameters B, C, and Δ as the function of the current input $x_{t}$ is the main innovation of Mamba, so as to realize the dynamic dependence of parameters on the input content. This selective mechanism allows the model to selectively integrate the historical state according to the current input to realize content perception reasoning, as shown in the equation (5):

(5) $${\bar{B}}_{t}=f_{B}\left(x_{t}\right),\;{\bar{C}}_{t}=f_{C}\left(x_{t}\right),\;{\text{Δ}}_{t}=f_{\text{Δ}}\left(x_{t}\right)$$

**Figure 1:**
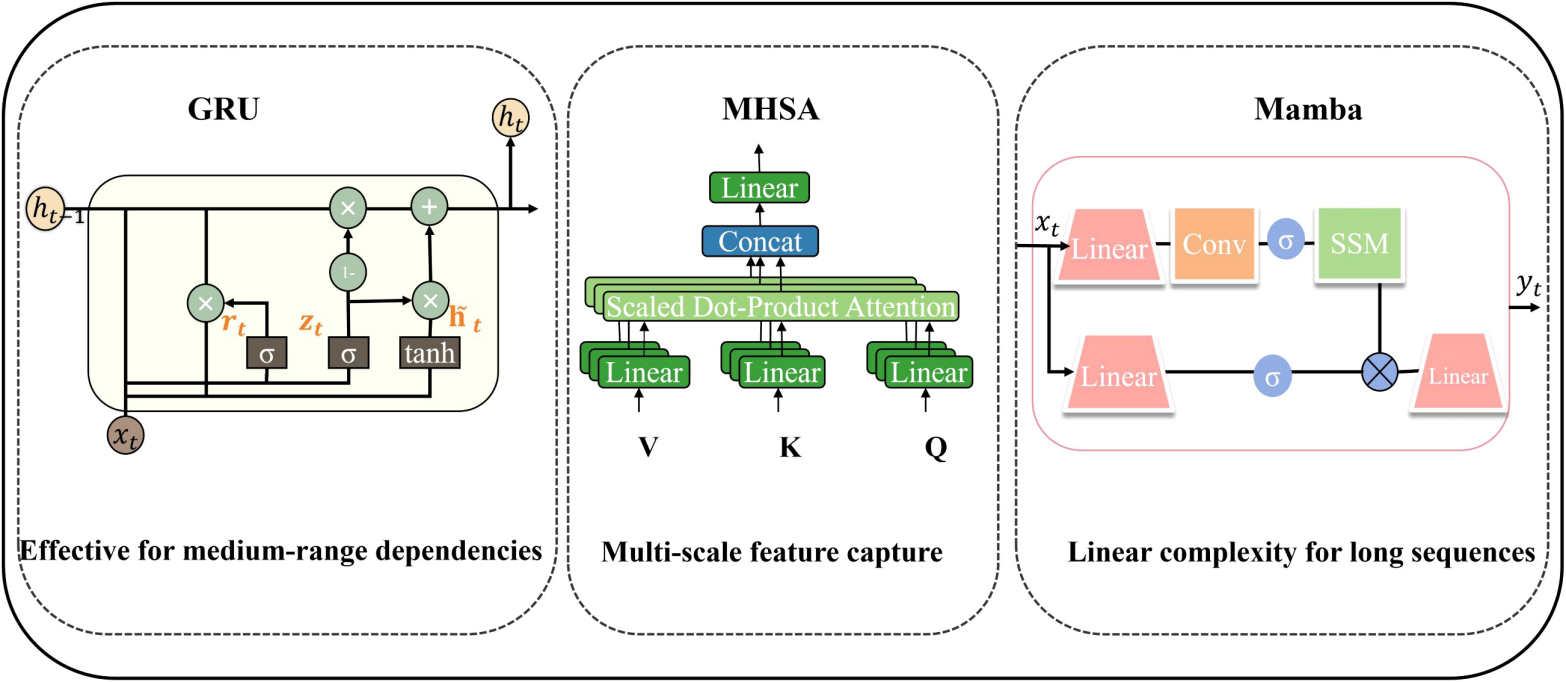
Temporal modeling methods.

Mamba further enhances SSMs by introducing a selective state space mechanism that enables content-aware reasoning while maintaining computational efficiency. Mamba uses hardware perception algorithms to optimize computing efficiency. This method significantly accelerates the execution of the model on modern hardware by parallelizing and integrating key computing kernels. Additionally, it dynamically manages intermediate results in memory through a strategic combination of storage and selective recomputation. The structure of Mamba (Gu and Dao, 2024) is illustrated in **Figure 1**.

2. GRU: GRU (Wei et al., 2021) provides a simple and effective implementation for RNN. Compared with traditional LSTM, it achieves competitive performance with fewer parameters and lower computational overhead. GRU effectively captures the temporal dynamic characteristics of the sequence through its gate control mechanism, and regulates the flow of information through selective retention and discarding of relevant states. **Figure 1** depicts the general structure of GRU. Reset gate ${\text{r}}_{t}$ and update gate ${\text{z}}_{t}$ are two gates that make up GRU. Here, ${\text{r}}_{t}$ governs the integration of new input with prior memory, and modulates information flow from previous hidden state $h_{t-1}$ into current candidate state ${\tilde{h}}_{t}$. Meanwhile, $z_{t}$ regulates information flow from previous hidden state $h_{t-1}$ to current hidden state $h_{t}$. At each time step *t*, given $x_{t}$ and $h_{t-1}$, $z_{t}$and $r_{t}$ are calculated as shown in the equation (6):

(6) $$z_{t}=\sigma \left({\text{W}}_{z}x_{t}+{\text{U}}_{z}h_{t-1}\right)\;r_{t}=\sigma \left({\text{W}}_{r}x_{t}+{\text{U}}_{r}h_{t-1}\right)$$

where *σ* denotes sigmoid function, which compresses the gate values into the interval [0, 1]. W and U stand for two learnable parameter matrices. The calculation formula for the candidate state ${\tilde{h}}_{t}$ is as shown in the equation (7).

(7) $${\tilde{h}}_{t}=\tanh\;\left({\text{W}}_{\tilde{h}}x_{t}+U_{\tilde{h}}\left(r_{t}⊙h_{t-1}\right)\right)$$

Here, ⊙ represents element-wise multiplication. Subsequently, the update gate determines how to combine old state $h_{t-1}$ and the candidate state ${\tilde{h}}_{t}$ to form current final state $h_{t}$, as shown in the equation (8):

(8) $$h_{t}=\left(1-z_{t}\right)⊙h_{t-1}+z_{t}⊙{\tilde{h}}_{t}\;{\text{y}}_{t}=\sigma \left({\text{W}}_{o}h_{t}\right)$$

3. Multi-head self-attention: MHSA (Vaswani et al., 2017) excels at concurrently attending to distinct representation subspaces across multiple positions, thereby capturing a wide spectrum of dependencies within a single layer. MHSA works by running several self-attention heads side by side. Each uses the Scaled Dot-Product Attention (SDPA) to deal with its own characteristic relationship. More specifically: First, the input sequence undergoes several different linear transformations to produce a separate set of query, key, and value vectors, and each vector is aligned with the same dimension. It then computes attention weight matrices via similarity measurements between the query and key vectors, which are used to perform weighted aggregation of the value vectors. Finally, the outputs from all attention heads are concatenated and fused, yielding a final representation that integrates multi-perspective information. **Figure 1** shows MHSA’s detailed network structure (Vaswani et al., 2017).

For an input sequence *x*, it undergoes *ℎ* linear transformations to derive the matrices Q, K, and V, as shown in the equation (9).

(9) $$Q=xW^{Q},\;K=xW^{K},\;V=xW^{V}$$

where learnable projection matrices are denoted by $W^{Q}$, $W^{K}$, and $W^{Z}$. Here is how the SDPA is calculated, as shown in the equation (10):

(10) $$\text{SelfAttention}\left(Q,K,V\right)=\text{softmax}\left(\frac{QK^{T}}{\sqrt{d_{k}}}\right)V$$

where $d_{k}$ denotes the dimension of the key vectors K.

Multi-head self-attention executes this process in parallel *ℎ* times (i.e., with *ℎ* heads), with each head learning feature relationships in a distinct subspace. The outputs are aggregated by concatenation and subsequently undergo a linear projection through matrix ${\text{W}}^{O}$.

(11) $$\text{MultiHead}\left(x\right)=\text{Concat}\left({\text{head}}_{1},\ldots ,{\text{head}}_{h}\right){\text{W}}^{O}$$

where ${\text{head}}_{i}=\text{Attention}\left({\text{QW}}_{i}^{Q},{\text{KW}}_{i}^{K},{\text{VW}}_{i}^{V}\right),\;i=1,\ldots ,h$.

#### 2.1.2 Limitations of temporal modeling methods

Although GRU, MHSA and Mamba are widely used in timing modeling, they still have inherent defects when applied to flight fault diagnosis. GRU and its Bidirectional GRU (BiGRU) (Chen et al., 2019) effectively captures local dependencies through the gate control mechanism, but its serial structure has a long-term information attenuation problem, and the key fault characteristics will gradually weaken in a longer period of time. In addition, its parallel ability is limited, resulting in low computing efficiency under high sampling rate sequences. MHSA realizes global dependence modeling through parallel multi-subspace attention, but its quadral computing complexity makes it difficult to deploy in a resource-limited environment. More importantly, MHSA is less sensitive to local transient details, often ignores the weak fault fluctuations in the early days, and its high dependence on the amount of data makes it easy to over-fit in low-sample scenarios. Mamba realizes the dynamic modeling of content perception through state conversion related to input, which has advantages in information filtering and efficiency. However, it is still biased toward local context, has limited modeling ability for ultra-long global dependence, and lacks a multi-scale feature extraction mechanism, and cannot capture high-frequency transient signals and low-frequency trends at the same time. Therefore, a single mechanism cannot meet the comprehensive requirements of local sensitivity, global perception ability and computing efficiency at the same time. A complementary architecture is urgently needed to integrate the advantages of various mechanisms to overcome their respective shortcomings.

### 2.2 Model design

**Figure 2:**
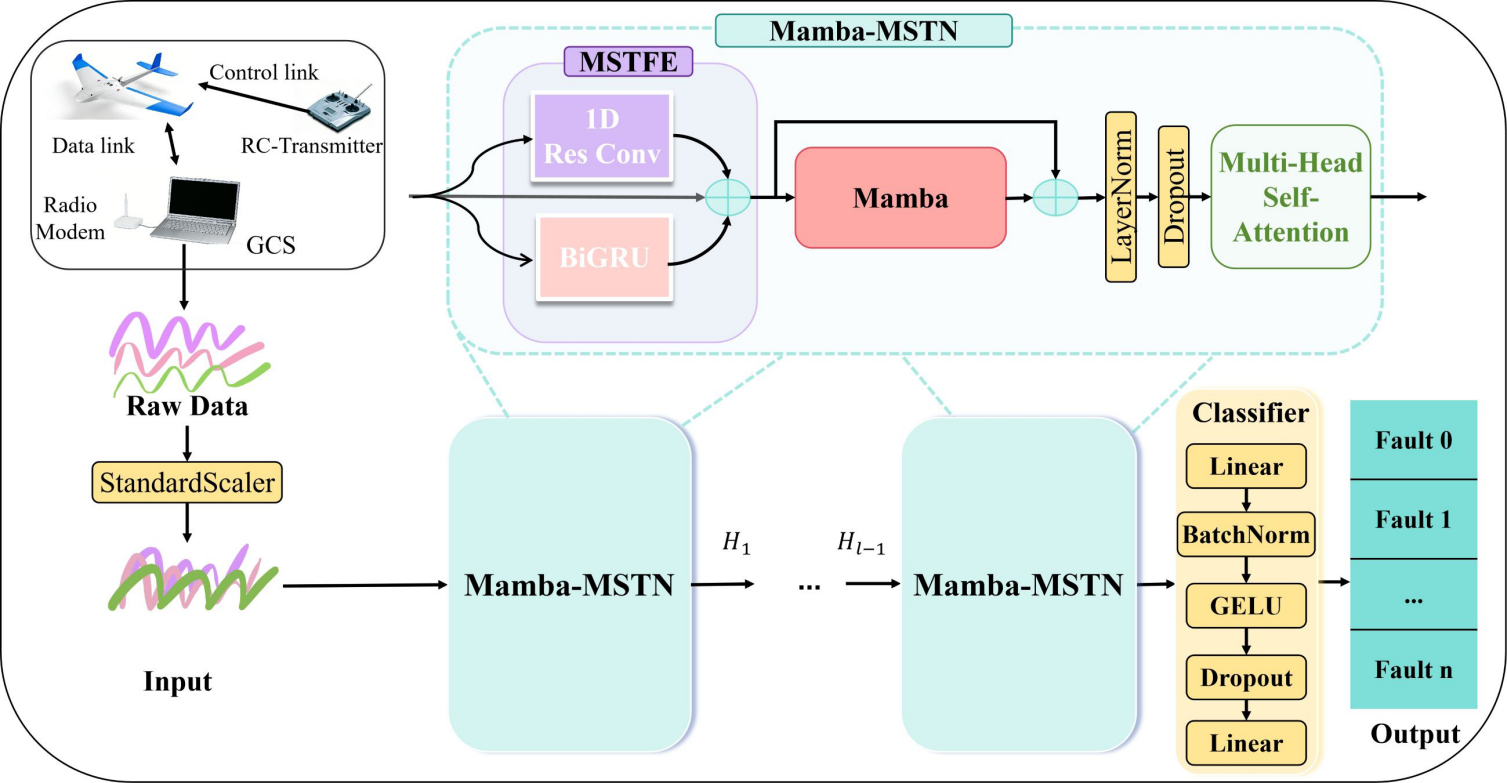
Overall framework of Mamba-MSTN.

The proposed sequence of MSTFE, Mamba and MHSA aims to establish a progressive refinement pipeline to convert the original flight data into a robust representation under low sample constraints. 1DRCNN and BiGRU are first used in the MSTFE module to extract multi-granular characteristics and capture the transient details and long-term evolutionary trends of fine-grained. Together, they produce rich but unfiltered characteristic spaces, which contain fine-grained fluctuations and evolutionary trends. However, the resulting feature space may contain redundancy; therefore, Mamba is introduced as an adaptive perception module, which is based on input-related state conversion dynamic screening and compression features. The processed output is now both exquisite and compact, which can be used as an ideal input for the final MHSA module, which establishes a global dependency representation through parallel multi-subspace attention. By operating on the screened features, MHSA can pay more attention to the critical time period without being overwhelmed by noise or redundancy, thus compensating for the local deviation inherent in the previous module. In this cascade architecture, each module not only completes its designated functions, but also gradually improves the representation quality passed to the next module to ensure collaboration to achieve accurate and efficient fault diagnosis. In this work, we propose the Mamba-MSTN framework to systematically enhance the processing of flight time-series data by improving multi-scale feature extraction, adaptive state selection, and global information fusion. **Figure 2** shows general design of Mamba-MSTN. The number of stacked blocks in Mamba-MSTN varies for low-sample fault diagnosis tasks.

The Mamba-MSTN framework primarily consists of the following three significant modules.

#### 2.2.1 MSTFE module

MSTFE module adopts a parallel dual-path architecture comprising 1D-RCNN and BiGRU. The residual convolution path employs multi-scale convolutional kernel to precisely capture transient detailed features within the flight data. Meanwhile, the BiGRU path models and extracts long-term evolutionary trends and contextual dependencies of sequential data from both forward and backward directions. The outputs from both paths, along with the original input, undergo feature fusion to generate multi-granularity temporal representations that integrate transient details and long-term trends. This provides rich and complementary feature support for subsequent state modeling.

The implementation details of this module are as follows.

**Figure 3:**
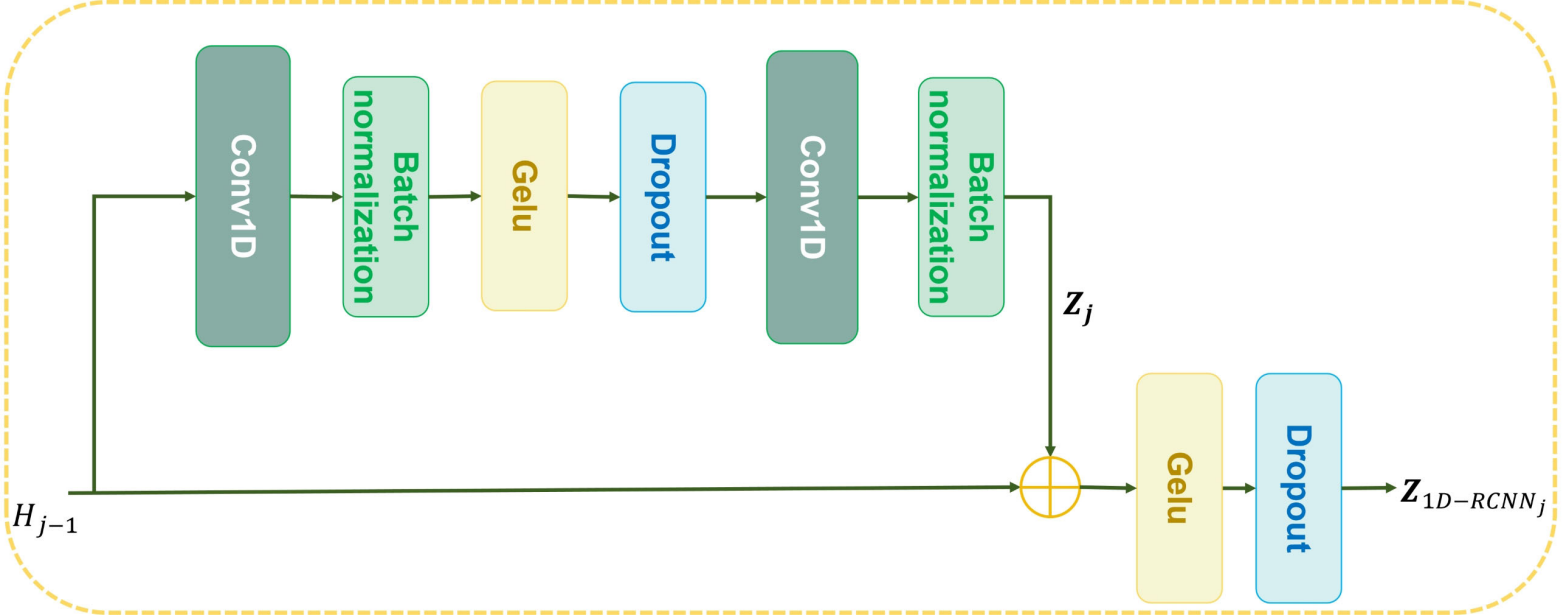
Overall 1D-RCNN structure.

1. 1D-RCNN: **Figure 3** illustrates structure of 1D-RCNN. Specifically, two convolutional operations with identical configurations and residual connection are employed. Assume that input is represented as
2. BiGRU: BiGRU stacks one GRU that runs forward in time with another that runs backward. Together, they capture patterns from both past and future states at once. This design helps pull richer features from the sequence, which makes the model stronger at handling time-based data. The mathematical expression is as follows: ${\text{h}}_{t}^{bi}=\left[{\text{h}}_{t}^{f},{\text{h}}_{t}^{b}\right]$, where ${\text{h}}_{t}^{f}$ and ${\text{h}}_{t}^{b}$ are forward GRU output and backward GRU output, respectively. The output of the BiGRU block is ${\text{Z}}_{{\text{BiGRU}}_{j}}$. $H_{j-1}\in {\mathbb{R}}^{B\times L\times D_{h}}$, where j represents the j −th Mamba-MSTN block (j = 1, . . . , l), B, L and $D_{h}$ stand for batch size, sequence length and hidden feature dimension. 1D-RCNN’s output is ${\text{Z}}_{1\text{D}-{\text{RCNN}}_{j}}$.

The output of the MSTFE module is obtained by integrating local features of the 1D-RCNN Block branch, the time-dependent characteristics of the BiGRU branch and the original feature $H_{j-1}$ by element:

(12) $${\text{MSTFE}}_{{\text{out}}_{j}}={\text{Z}}_{1\text{D}-{\text{RCNN}}_{j}}+{\text{Z}}_{{\text{BiGRU}}_{j}}+H_{j-1}$$

#### 2.2.2 Mamba-based adaptive state perception and screening module

In order to further improve modeling efficiency and dynamic perception ability of long sequences, we introduce Mamba as the core component of state screening and sequence modeling. Through its input-dependent selective scanning mechanism, Mamba dynamically allocates weights and filters information across multi-scale temporal features. This adaptive mechanism directs the model’s attention to meaningful states related to the current input while reducing the interference of redundant information. The module models remote dependencies with linear time complexity, which significantly improves model sensitivity for state tracking and anomaly detection in dynamic flight data changes.

The input ${\text{MSTFE}}_{{\text{out}}_{j}}$ to Mamba module is processed through two parallel branches. Along one path, the sequence is linearly transformed and activated via SiLU to produce $x_{j}$. Along the other, the input is first linearly projected and then fed into a one-dimensional convolution, and $y_{j}$ is activated by SiLU. Then, $y_{j}$ enters SSM for sequence modeling, producing output $\hat{y_{j}}=SSM_{\theta \left(MSTFE_{out_{j}}\right)}\left(y_{j}\right)$, where SSMθ (·) represents the SSM's core parameter θ, including, for instance, the discrete matrix A and the projection matrices B and C. These parameters are dynamically generated by a linear layer within the Mamba block from the input ${\text{MSTFE}}_{{\text{out}}_{j}}$, so as to achieve selective sequence modeling. Finally, the final output is obtained by fusing the outputs $x_{j}$ and $\hat{y_{j}}$ from the two branches using element-wise multiplication and projecting them through a linear layer to restore target dimension, as shown in the equation (13).

(13) $${\text{Z}}_{{\text{Mamba}}_{j}}=\text{Linear}\left(x_{i}⊗\hat{y_{j}}\right)$$

where ⊗ indicates element-by-element multiplication.

#### 2.2.3 MHSA-based global dependency modeling module

To address potential shortcomings in capturing cross-variable and cross-timestep dependencies through local modeling alone, the framework incorporates the MHSA mechanism. It can accurately focus on fault-relevant segments within the data and effectively enhance the synergy across different feature dimensions. This leads to an improved ability to represent global temporal dependencies. By making up for the inherent limitations of local modeling, the model shows significantly improved performance in discriminating and accurately identifying complex fault patterns.

The output of Mamba module ${\text{Z}}_{{\text{Mamba}}_{j}}$ is added to ${\text{MSTFE}}_{{\text{out}}_{j}}$, and then the layer normalization and dropout regularization are carried out to form the input of the MHSA module, which is written as ${\text{Z}}_{{\text{MHSA}}_{in_{j}}}$.

The output of the j−th Mamba-MSTN block is written as Hj, and it goes straight into the next block as its input. H is the output from the last block. At the end, a simple classifier turns the high level features into a category label.

In summary, the proposed Mamba-MSTN framework establishes a hierarchical and progressive learning pathway for fault features. This pathway originates from the MSTFE module, which collaboratively extracts both transient details and long-term evolutionary trends to construct a foundational multi-granularity temporal representation. Subsequently, the Mamba-based adaptive state perception and screening module takes the features output from the previous stage as its input. Its selective states-pace mechanism enables content-aware modeling of temporal dynamics, dynamically screening and emphasizing key state information that is highly relevant to faults. Finally, the MHSA module globally interacts with and fuses the screened features across timesteps and subspaces, while adaptively focusing on key segments. These three components are organically integrated and complementary, providing comprehensive and efficient feature support and modeling capabilities for the fault diagnosis of complex flight systems.

### 2.3 Dataset description

**Figure 4:**
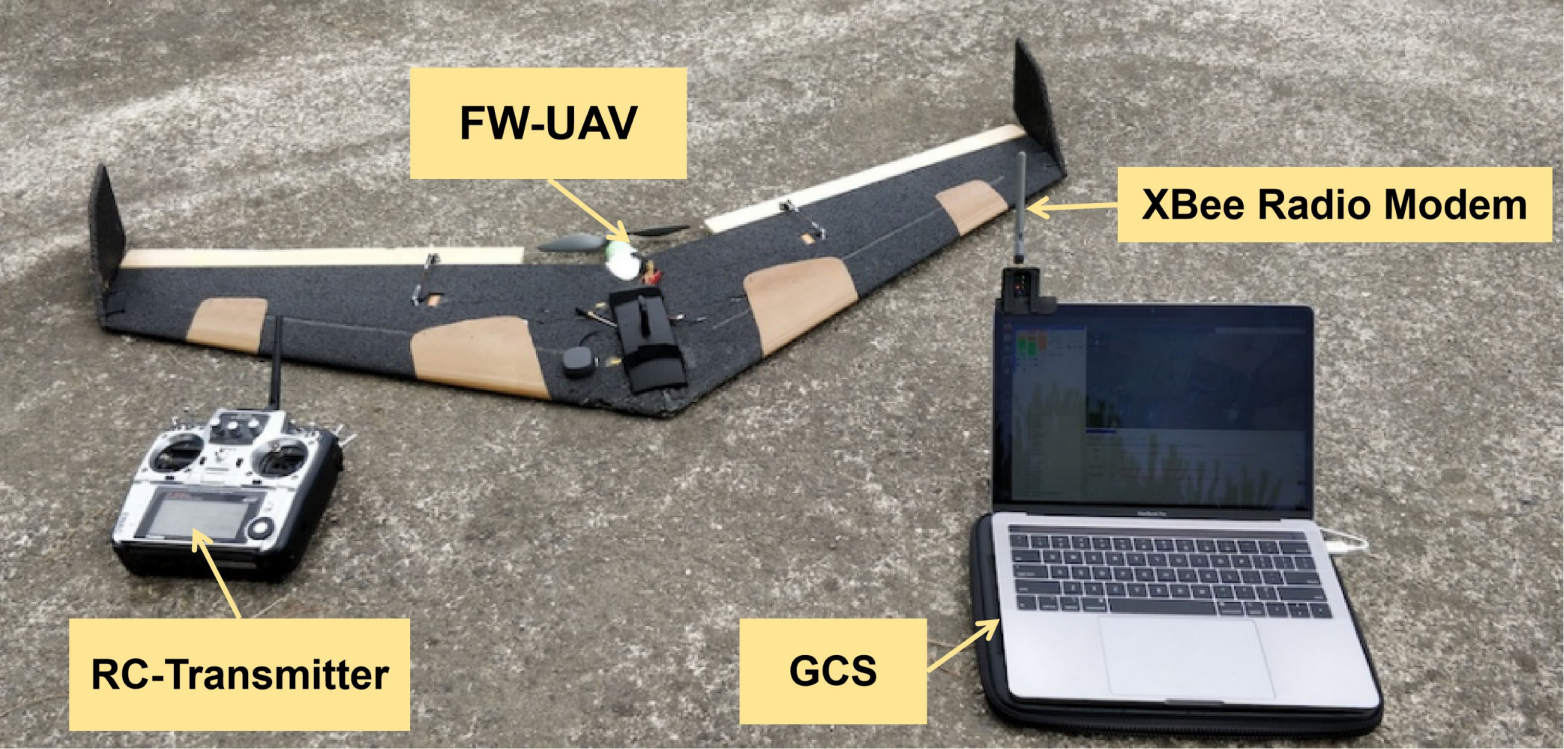
Data acquisition systems.

This article uses the public data set of UAVs failures released by Bronz et al. (Bronz et al., 2020). **Table 1** and **Figure 4** show flight test system’s specifications. Flight system is primarily divided into three core modules: the UAVs airframe, the remote control transmitter (RC-Transmitter), and the ground control station (GCS).

**Table 1:** Technical description of flight system.

Component	Metric	Component	Metric
Wing extent	1.2 m	Flight duration	60 min
Surface area	0.28 m^2^	GPS model	U-Blox M8N
Weight	0.75 kg	Motor	T-Motor 2208/18-Kv 1100
Battery backup	30 Wh	Autopilot	Paparazzi Chimera v1.0
Flight acceleration	12 m/s		

To get the required experimental data, fault commands were sent from a ground station while the fixed wing UAVs was being flown manually via radio control. This gave us flight records from several different days. For this study, we follow the same selection process as in reference (Bronz et al., 2020) and pick comparable datasets from July 12, 13, 21, and 23. **Table 2** summarizes the flight data under different failure types. The July 12 and 13 data contain only two conditions: normal and fault. In the fault case, the parameter *d*_1_ = 0.3 means the right-wing control surface only had 30% of its normal effectiveness. Data from July 21 and July 23, however, cover nine failure types. Seven of these faults were created by reducing the left-wing control surfaces effectiveness gradually from 0.9 down to 0.3, while the other two faults were introduced differently.

**Table 2:** Dataset specifics.

Dataset date	Wind speed	Categories	Fault type
July 12	*<* 2.0 m/s	2	Normal, $d_{1}$ = 0.3
July 13	8.0 m/s	2	Normal, $d_{1}$ = 0.3
July 21	2.5 m/s	9	Normal, $d_{1}$ = 0.3, $d_{2}$ = 0.9 ∼ 0.3
July 23	5.0 m/s	9	Normal, $d_{1}$ = 0.3, $d_{2}$= 0.9 ∼ 0.3

### 2.4 Experimental configuration and setup

This study’s experimental setup is as follows: the server is equipped with dual Intel Xeon Gold 6430 processors, 512 GB DDR5 memory, and 8 NVIDIA RTX A6000 professional graphics cards. The system is also configured with CUDA 11.8 and PyTorch 2.3.1.

The original flight data is preprocessed to ensure calculation efficiency and the integrity of the model input. The original data contains more than 50 flight variables. In order to focus on the core dynamic characteristics and reduce the computational load, according to (Huang et al., 2024), we selected 12 key variables in this paper: airspeed (v), attitude angles (yaw ψ, pitch θ, roll ϕ), triaxial linear acceleration $\left(a_{x},\;a_{y},a_{z}\right)$, triaxial angular velocity $\left(w_{x},\;w_{y},w_{z}\right)$, and control inputs $\left(u_{1},\;u_{2}\right)$.

We assessed our framework in real-world low-sample scenarios to evaluate its UAVs fault classification ability. Tests included both binary and multi-class tasks, conducted with varying amounts of training data. The experimental data comprised four flight recordings, acquired on July 12, 13, 21, and 23, 2020, the total daily sample sizes were 8,980, 8,980, 21,980, and 21,980, respectively. To analyze the impact of different data scales on model performance, we randomly extracted multiple gradient-level subsamples from the daily data, with specific sample sizes set at 90, 120, 150, 180, 270, 360, 720, and 1,440. According to (Zhang et al., 2024), for the subsamples of the daily flight data, we used 70% of each subsample as the training set to update model parameters, while the remaining 30% served as a validation subset to monitor training dynamics and retain the optimal model. Taking 90 samples extracted from the flight data of July 12 as an example, we used 63 samples for model training and held out the remaining 27 for validation. Additionally, 10% of samples are randomly chosen from the total flight data as an independent test set to thoroughly assess the model’s generalization capability. The test sample sizes for July 12, 13, 21, and 23 are 898, 898, 2198, and 2198, respectively. During training, we use a batch size of 16, a learning rate of 0.001, and a dropout rate of 0.3. We conducted ten repeated experiments and calculated the average accuracy as the final result. The Mamba–MSTN block configuration for the July 12, 13, 21, and 23 datasets is set to 1 block, 1 block, 5 blocks, and 3 blocks, respectively. **Table 3** shows the specific experimental parameter settings.

**Table 3:** Parameter configuration of Mamba-MSTN.

Module	Sub-module	Key parameters	Output dimension
MSTFE	1D-RCNN	in_channels=128, out_channels=128, kernel_size=5, stride=1, padding=2	(B, L, 128)
	BiGRU	input_size=128, hidden_size=64, num_layers=2	(B, L, 128)
Mamba	Selective SSM	model_dim=128, state_dim=32, conv_kernel=4, expansion=2	(B, L, 128)
MHSA	MHSA	embed_dim=128, num_heads=4	(B, L, 128)
Classifier	Linear_1_	in_features=128, out_features=64	(B, 64)
	Linear_2_	in_features=64, out_features= N	(B, N)

B stands for batch size, L is the sequence length, and N is the number of fault categories.

## 3 Results

### 3.1 Diagnosis result analysis

**Figure 5:**
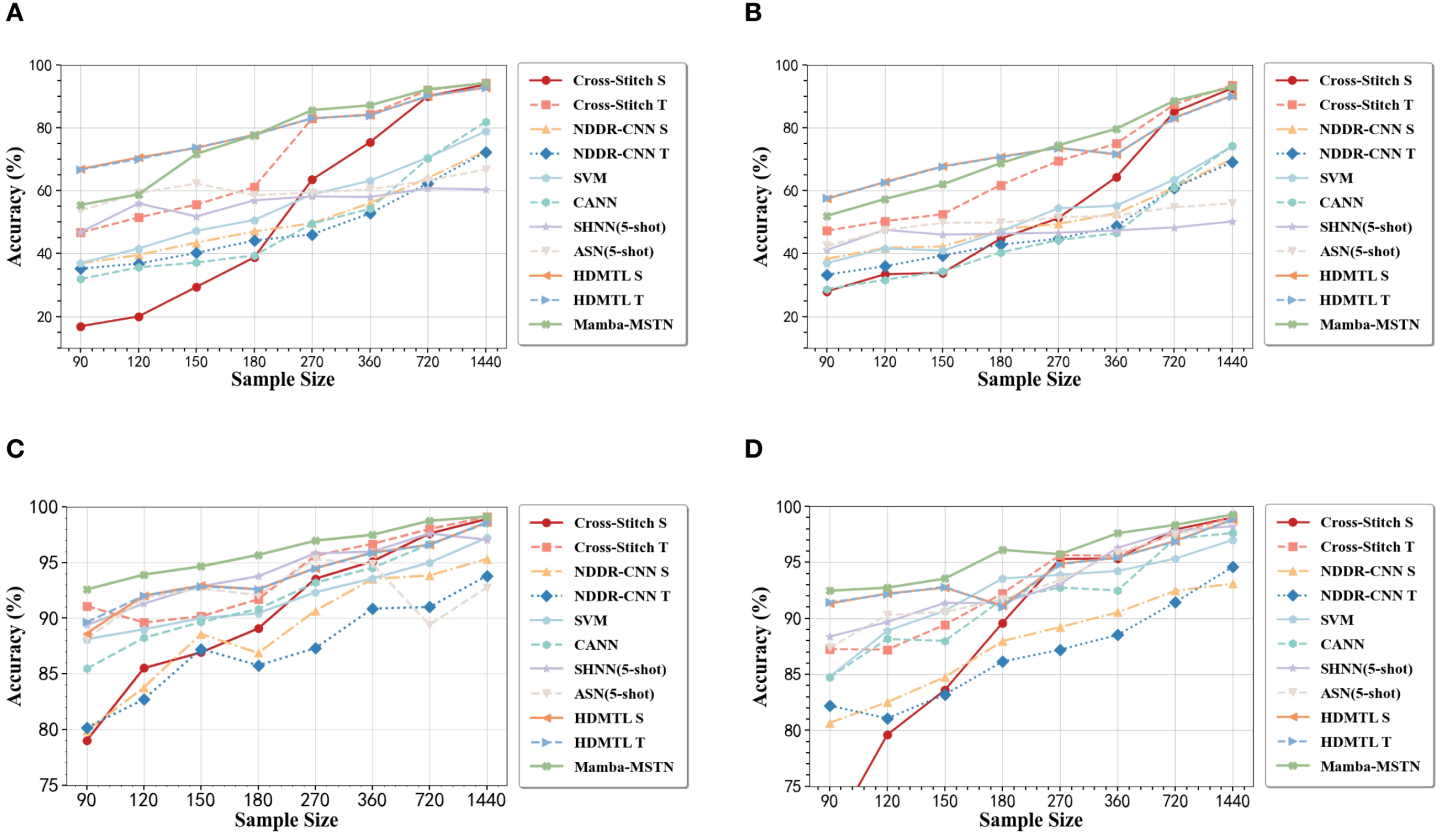
Visual comparison of diagnostic accuracy (%) among models under different sample sizes (panels **(a)–(d)** correspond to experimental comparisons conducted on July 12, 13, 21, and 23, respectively).

We compared our method against several baselines, including SVM, meta-learning, and single-task learning approaches. The baselines include Cross-Stitch (Misra et al., 2016), NDDR-CNN (Gao et al., 2019), SVM (Bronz et al., 2020), CANN (Tran et al., 2021), SHNN (Li et al., 2022), ASN (Xiong et al., 2023) and HDMTL (Huang et al., 2024). **Tables 4**-**7** show the fault diagnosis results of July 12, 13, 21 and 23 correspondingly. **Figure 5** visualizes the diagnosis results. The top result is bolded.

**Table 4:** Accuracy (%) in binary classification task (July 12).

Models		Sample size							
		90	120	150	180	270	360	720	1440
Cross-Stitch	S	79.01	85.51	86.92	89.07	93.54	95.10	97.61	98.90
	T	91.09	89.61	90.16	91.70	95.61	96.68	98.00	99.11
NDDR-CNN	S	79.87	83.78	88.58	86.87	90.67	93.52	93.84	95.32
	T	80.17	82.71	87.23	85.76	87.30	90.86	91.00	93.80
SVM		88.11	89.00	90.00	90.44	92.33	93.56	95.00	97.22
CANN		85.46	88.23	89.65	90.83	93.19	94.52	96.64	98.53
SHNN (5-shot)		89.38	91.31	92.87	93.76	95.82	95.98	97.61	97.04
ASN (5-shot)		88.16	91.82	92.68	92.06	95.30	94.86	89.40	92.76
HDMTL	S	88.64	92.03	92.93	92.60	94.49	95.89	96.60	98.61
	T	89.64	92.01	92.85	92.60	94.49	95.86	96.58	98.61
Mamba-MSTN		**92.58**	**93.91**	**94.65**	**95.68**	**96.96**	**97.49**	**98.75**	**99.13**

Best results are bolded.

**Table 5:** Accuracy (%) in binary classification task (July 13).

Models		Sample size							
		90	120	150	180	270	360	720	1440
Cross-Stitch	S	70.40	79.59	83.59	89.58	95.29	95.35	97.93	99.00
	T	87.25	87.20	89.42	92.22	95.63	95.58	97.60	99.02
NDDR-CNN	S	80.66	82.50	84.75	87.96	89.21	90.53	92.45	93.09
	T	82.21	81.06	83.18	86.13	87.20	88.51	91.43	94.61
SVM		84.76	88.88	90.66	93.55	93.88	94.22	95.33	97.00
CANN		84.72	88.15	87.98	91.69	92.74	92.49	97.10	97.62
SHNN (5-shot)		88.36	89.66	91.37	91.35	93.05	96.30	97.80	98.22
ASN (5-shot)		87.37	90.30	90.56	91.69	93.39	95.86	97.48	98.63
HDMTL	S	91.31	92.21	92.73	91.13	94.87	95.43	96.91	98.82
	T	91.42	92.14	92.81	91.00	94.81	95.43	96.84	98.85
Mamba-MSTN		**92.46**	**92.73**	**93.54**	**96.11**	**96.35**	**97.59**	**98.34**	**99.27**

Best results are bolded.

**Table 6:** Accuracy (%) in multi-classification task (July 21).

Models		Sample size							
		90	120	150	180	270	360	720	1440
Cross-Stitch	S	16.88	19.98	29.36	38.74	63.48	75.34	89.98	93.70
	T	46.63	51.45	55.52	61.13	82.83	84.19	91.90	94.23
NDDR-CNN	S	36.87	39.53	43.44	46.99	49.60	56.02	63.98	72.61
	T	35.17	36.79	40.17	44.12	46.06	52.73	62.27	72.19
SVM		36.96	41.58	47.18	50.64	58.76	63.12	70.48	78.79
CANN		31.87	35.59	37.10	39.38	49.46	54.24	70.13	81.83
SHNN (5-shot)		46.80	55.91	51.77	56.88	58.15	57.96	60.69	60.35
ASN (5-shot)		53.65	59.18	62.22	58.49	59.35	60.35	63.22	66.67
HDMTL	S	**66.83**	**70.50**	73.53	**77.76**	82.94	84.06	89.97	92.67
	T	66.65	70.02	**73.69**	77.63	83.01	83.91	90.04	92.77
Mamba-MSTN		55.94	63.04	68.67	74.97	**85.18**	**87.39**	**92.37**	**94.84**

Best results are bolded.

**Table 7:** Accuracy (%) in multi-classification task (July 23).

Models		Sample size							
		90	120	150	180	270	360	720	1440
Cross-Stitch	S	27.96	33.39	33.85	44.68	51.25	64.27	84.94	92.47
	T	47.26	50.25	52.49	61.76	69.39	74.89	87.30	**93.44**
NDDR-CNN	S	38.25	41.77	42.24	47.64	49.44	52.74	60.87	69.98
	T	33.30	35.93	39.35	42.84	44.69	48.69	60.80	68.98
SVM		36.96	41.58	40.92	46.96	54.38	55.18	63.48	74.01
CANN		28.66	31.64	34.29	40.32	44.25	46.44	60.99	74.27
SHNN (5-shot)		41.14	47.50	45.99	46.26	46.60	47.32	48.28	50.14
ASN (5-shot)		42.40	47.36	49.76	49.78	51.48	52.06	54.65	55.96
HDMTL	S	57.37	**62.71**	**67.71**	**70.74**	73.56	71.51	83.07	90.20
	T	**57.59**	62.54	67.61	70.61	73.54	71.64	82.99	90.00
Mamba-MSTN		51.96	57.31	61.96	68.70	**75.24**	**79.63**	**88.50**	92.88

Best results are bolded.

Judging from the binary classification results given in **Tables 4** and **5**, compared with existing models, the proposed method shows excellent accuracy across all test sample sizes. Specifically, in **Table 4**, as the sample size increased from 90 to 360, the accuracy increased by 0.81% to 1.92% compared with the best previously reported results. When the sample size was 180, the improvement peaked at 1.92%. As the sample size further expanded to 720 and 1440, the increase gradually narrowed. In **Table 5**, at smaller sample sizes from 90 to 180, the model usually shows a high improvement of 0.52% to 2.56%, indicating that it can effectively extract critical fault characteristics from limited data. When the sample size continues to increase to 270 and above, although there are certain fluctuations in the improvement of model performance, the overall stability is within the range of 0.25% to 1.29%. In terms of overall average performance, Mamba-MSTN has achieved the highest average accuracy rate in **Tables 4** and **5**, significantly better than all other comparison models, verifying its robust generalization ability under different sample sizes. The results show that in the binary classification task, the proposed architecture shows better generalization performance and feature extraction efficiency at different data scales.

**Tables 6** and **7** give the experimental results of multi-classification tasks. In most samples, the accuracy of the method proposed in this paper is higher than that of the existing model. As shown in **Table 6**, when the sample size exceeds 180, the method in this article is obviously better than the current mainstream method. In the range of sample size from 150 to 1440, the proposed framework continues to be better than most baseline methods, with an accuracy rate of 92.37% at 720 samples and 94.84% at 1440 samples, even higher than top-performing methods such as HDMTL and Cross-Stitch. This shows that although the performance is slightly inferior to HDMTL at a very small sample size (90 and 120 samples), it is still better than traditional models such as SVM, CANN and ASN. **Table 7** further confirms that when the sample size exceeds 180, the proposed framework shows strong performance. When the sample volume reaches 360 or above, the accuracy rate increases to 79.63%, further increases to 88.50% at 720 samples, and 92.88% at 1440 samples. These results are significantly better than SVM, CANN, ASN, SHNN and NDDR-CNN, and are equivalent or slightly superior to Cross-Stitch and HDMTL. However, at a very small sample size (90 or 120 samples), the performance is significantly reduced. For example, the accuracy rate is only 51.96% at 90 samples, which is significantly lower than HDMTL and only slightly better than Cross-Stitch. Judging from the average accuracy rate under all sample sizes, Mamba-MSTN reached 77.80% in **Table 6** and 72.02% in **Table 7**, which is competitive and has the same outstanding performance as HDMTL. In general, the main advantage of this method is its strong timing mode capture ability, which can achieve reliable fault detection in medium to large sample volumes. How to solve the challenges related to the very small sample scenarios in multi-classification tasks is still an important direction for future work.

**Figure 6:**
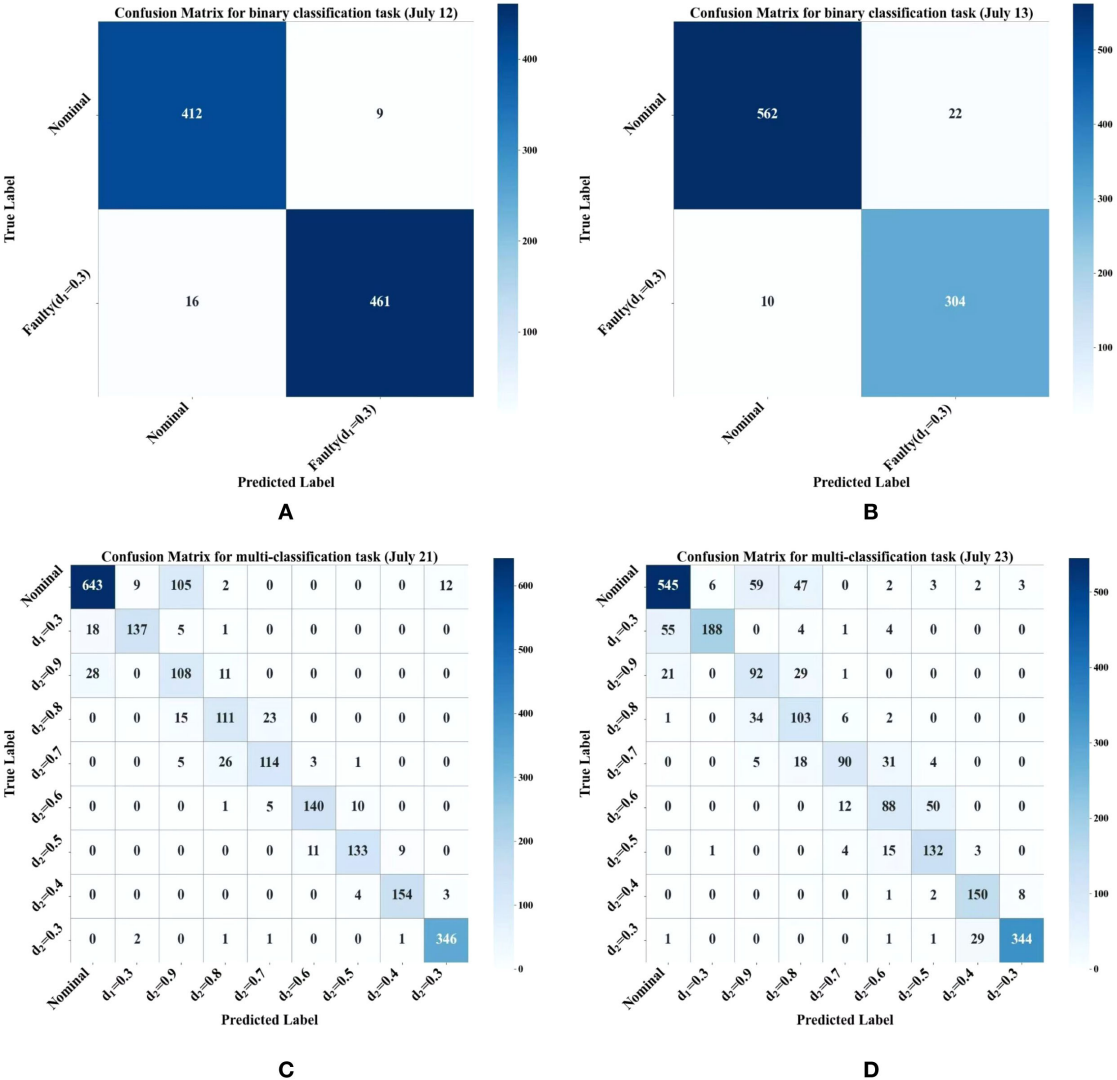
Confusion matrices with 270 sample size: **(a)** July 12, **(b)** July 13, **(c)** July 21, **(d)** July 23.

In order to further verify the validity of the proposed method, the prediction accuracy of different methods on four data test sets is compared under the 360 sample setting, as shown in **Table 8**. It can be seen that HDMTL and Cross-Stitch have achieved the best overall performance among all baseline methods, with an average accuracy of 86.72% and 87.84% respectively, representing the strongest baseline model. In this work, statistical significance tests are conducted between the proposed Mamba-MSTN and these two optimal baselines. The results demonstrate that Mamba-MSTN achieves a mean accuracy of 90.53%, which is significantly superior to both HDMTL and Cross-Stitch, while yielding the lowest standard deviation of 8.70%. The statistical test results confirm that the performance improvements of Mamba-MSTN over the state-of-the-art baselines are statistically significant, indicating its more reliable generalization ability and prediction robustness under small-sample conditions. **Tables 9**-**12** show the flight data classification reports when the sample size is 270. **Figure 6** shows the confusion matrix. The model performed excellently in binary classification tasks with minimal confusion, showing only slight false positives and false negatives. In multi-class tasks (July 21 and 23), performance demonstrates significant class dependency, with confusion primarily concentrated among mild faults and between normal and mild faults. Specifically, mild faults corresponding to *d*_2_ values of 0.9 and 0.8 yield the lowest F1-scores ranging from 0.56 to 0.74, characterized by extremely low precision but moderate recall, indicating that many samples from other classes are misclassified into these categories, effectively acting as confusion attractors. As the severity of the fault increases with the decrease of the *d*_2_ value, the feature severability increases accordingly. Under serious failures of *d*_2_ =0.4 and 0.3, the F1-scores exceeds 0.87, which ensures reliable identification effect. The leakage rate of the normal category is about 16%-18%, which is mainly mistakenly classified as a minor fault, which reflects the similarity between the normal state and the weak abnormality in terms of timing characteristics. It is worth noting that although categories with smaller sample sizes usually perform poorly, feature separability plays a more dominant role in model performance. For example, *d*_2_ = 0.4 despite the moderate sample size, excellent results have been achieved. In summary, the primary failure modes are confusion among mild faults and misclassifications between normal and mild faults. Future improvements should focus on enhancing feature separation for mild faults, for instance through contrastive learning or fine-grained feature extraction to amplify interclass differences.

**Table 8:** Performance comparison of different methods (360 sample size).

Models		July 12	July 13	July 21	July 23	Mean	Std
Cross-Stitch	S	95.10	95.35	75.34	64.27	82.52	15.36
	T	96.68	95.58	84.19	74.89	87.84	10.31
NDDR-CNN	S	93.52	90.53	56.02	52.74	73.20	21.80
	T	90.86	88.51	52.73	48.69	70.20	22.58
SVM		93.56	94.22	63.12	55.18	76.52	20.32
CANN		94.52	92.49	54.24	46.44	71.92	25.14
SHNN (5-shot)		95.98	96.30	57.96	47.32	74.39	25.49
ASN (5-shot)		94.86	95.86	60.35	52.06	75.78	22.86
HDMTL	S	95.89	95.43	84.06	71.51	86.72	11.52
	T	95.86	95.43	83.91	71.64	86.71	11.47
Mamba-MSTN		**97.49**	**97.59**	**87.39**	**79.63**	**90.53**	**8.70**

**Table 9:** July 12 flight binary classification report.

Metrics	Precision	Recall	F1-Score	Support
Nominal	0.9626	0.9786	0.9706	421
Faulty $\left(d_{1}\;=\;0.3\right)$	0.9809	0.9665	0.9736	477
accuracy			0.9722	898
macro avg	0.9717	0.9725	0.9721	898
weighted avg	0.9723	0.9722	0.9722	898

**Table 10:** July 13 flight binary classification report.

Metrics	Precision	Recall	F1-Score	Support
Nominal	0.9825	0.9623	0.9723	584
Faulty $\left(d_{1}\;=\;0.3\right)$	0.9325	0.9682	0.9500	314
accuracy			0.9644	898
macro avg	0.9575	0.9652	0.9612	898
weighted avg	0.9650	0.9644	0.9645	898

**Table 11:** July 21 flight multi-classification report.

Metrics	Precision	Recall	F1-Score	Support
Nominal	0.9332	0.8340	0.8808	771
$d_{1}\;=\;0.3$	0.9257	0.8509	0.8867	161
$d_{2}\;=\;0.9$	0.4538	0.7347	0.5610	147
$d_{2}\;=\;0.8$	0.7255	0.7450	0.7351	149
$d_{2}\;=\;0.7$	0.7972	0.7651	0.7808	149
$d_{2}\;=\;0.6$	0.9091	0.8974	0.9032	156
$d_{2}\;=\;0.5$	0.8986	0.8693	0.8837	153
$d_{2}\;=\;0.4$	0.9390	0.9565	0.9477	161
$d_{2}\;=\;0.3$	0.9584	0.9858	0.9719	351
accuracy			0.8581	2198
macro avg	0.8378	0.8487	0.8390	2198
weighted avg	0.8776	0.8581	0.8644	2198

**Table 12:** July 23 flight multi-classification report.

Metrics	Precision	Recall	F1-Score	Support
Nominal	0.8748	0.8171	0.8450	667
$d_{1}\;=\;0.3$	0.9641	0.7460	0.8412	252
$d_{2}\;=\;0.9$	0.4842	0.6434	0.5526	143
$d_{2}\;=\;0.8$	0.5124	0.7055	0.5937	146
$d_{2}\;=\;0.7$	0.7895	0.6081	0.6870	148
$d_{2}\;=\;0.6$	0.6111	0.5867	0.5986	150
$d_{2}\;=\;0.5$	0.6875	0.8516	0.7608	155
$d_{2}\;=\;0.4$	0.8152	0.9317	0.8696	161
$d_{2}\;=\;0.3$	0.9690	0.9149	0.9412	376
accuracy			0.7880	2198
macro avg	0.7453	0.7561	0.7433	2198
weighted avg	0.8104	0.7880	0.7937	2198

### 3.2 Efficiency analysis

As shown in **Table 13**, by analyzing the total parameter count of different methods and time required to diagnose a single sample in the fault detection task conducted on July 21, we demonstrate that our model is an efficient lightweight network, exhibiting significant competitive advantages.

**Table 13:** Diagnostic efficiency and parameter evaluation.

Models	Cross-stitch	NDDR-CNN	SVM	CANN	SHNN	ASN	HDMTL	Mamba-MSTN
Parameters	2.94×10^6^	4.32×10^6^	**198**	4.87×10^6^	3.85×10^4^	5.24×10^4^	1.28×10^7^	2.72×10^6^
Time consumed (s)	0.001676	0.001170	**0.000020**	0.001689	0.039216	0.038743	0.002286	0.000510

Best results are bolded.

In terms of running time, the reasoning time of this method is only 0.000510 seconds, which is the shortest among all deep learning methods involved in comparison. It is not only significantly better than methods with the same number of parameters, such as CANN, NDDR-CNN and Cross-Stitch, but also the reasoning speed is 3.3 times faster than the Cross-Stitch network with only 2.94 × 10^6^ parameters. More importantly, compared to the current high-performance heterogeneous multi-task learning model HDMTL, the proposed method reduces inference time by approximately 78%. These findings provide clear evidence that the proposed engineering deployment architecture has lower latency and greater real-time processing potential.

Regarding the parameter scale, the proposed method comprises 2.72 × 10^6^ parameters, which is in the upper-middle range among the comparative models. It is much lighter than the HDMTL model with more than 100 million parameters and smaller than certain CNN variants with about 4 million parameters. This shows that the performance achieved by the proposed method does not come from simple parameter stacking, but from more effective architecture design, which can achieve stronger feature extraction.

### 3.3 Hyperparameter sensitivity analysis

The primary hyperparameters include loss rate, batch size, quantity of MHSA heads, Mamba-MSTN blocks, hidden layers, and other related factors. Determine the best combination of these parameters to ensure the highest performance that can be achieved. Using different hyperparameter settings, 270 samples were used for experiments comparisons. **Table 14** presents the corresponding results.

**Table 14:** Performance comparison of different parameters.

Hyperparameter	Setting	July 12	July 13	July 21	July 23
Mamba-MSTN blocks	1	**96.96**	**96.35**	73.13	67.84
	3	96.25	95.92	79.23	**75.24**
	5	95.19	95.94	**85.18**	75.05
hidden dim	64	96.94	96.27	81.39	73.90
	128	**96.96**	**96.35**	**85.18**	**75.24**
	256	95.97	95.84	81.68	73.17
batch size	8	95.89	95.88	77.69	73.72
	16	**96.96**	**96.35**	**85.18**	**75.24**
	24	96.46	95.81	82.84	75.05
dropout	0.2	96.66	95.96	85.11	71.28
	0.3	**96.96**	**96.35**	**85.18**	**75.24**
	0.4	95.65	96.33	83.34	74.20

Best results are bolded.

### 3.4 Ablation study

**Figure 7:**
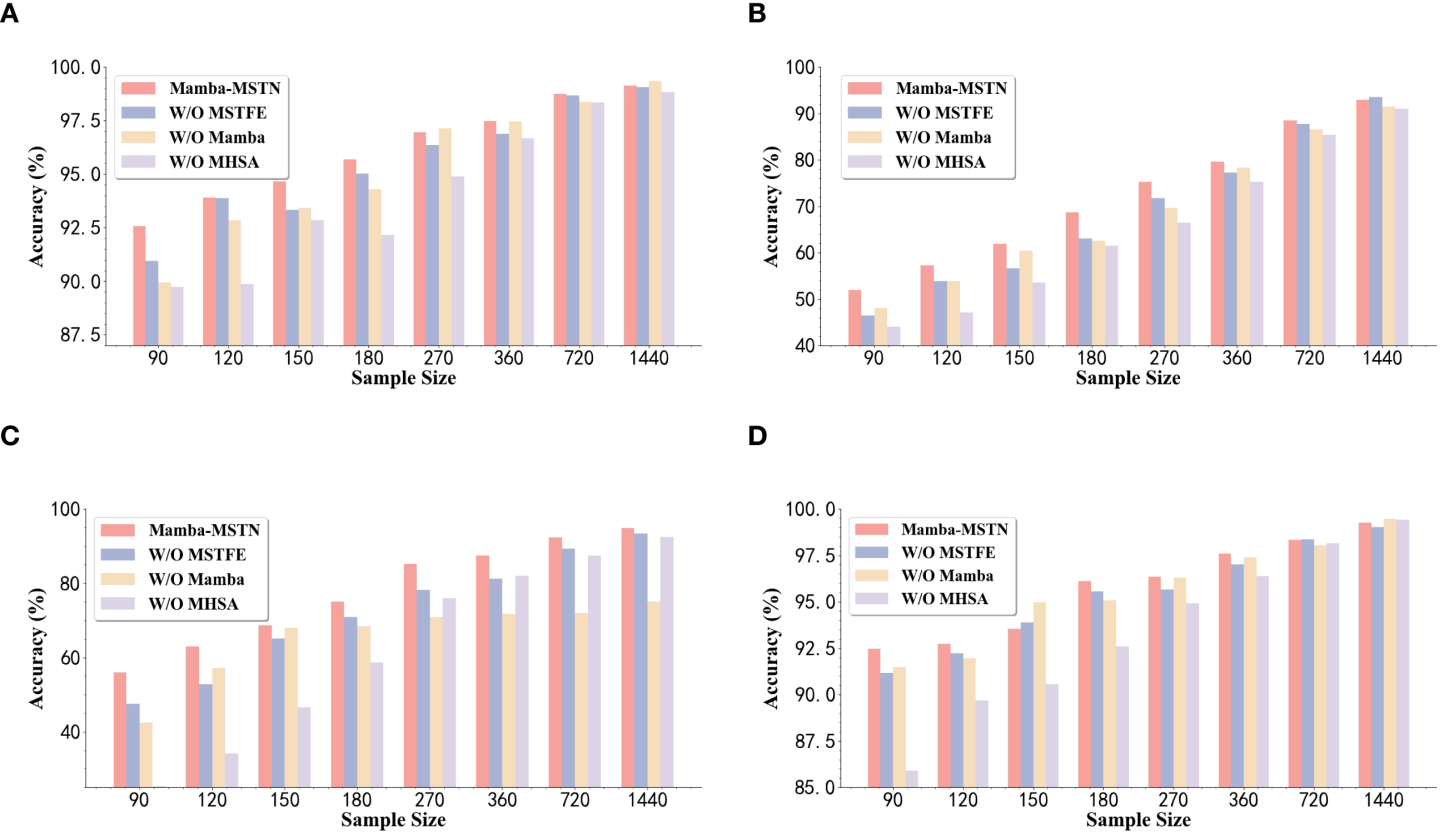
Visualization of ablation study comparison (panels **(a)–(d)** correspond to experimental comparisons conducted on July 12, 13, 21, and 23, respectively).

To assess how each component contributes to the overall framework performance, we conducted a model melting study. The first line reports the performance of the complete architecture. In each subsequent line, we exclude one functional module while keeping other functional modules unchanged. Specifically, the MSTFE module is deleted in the second line. The adaptive state perception and filtering module Mamba was deleted in the third line. Delete the global dependency modeling module MHSA in the last line. **Tables 15**-**18** show the results obtained from these configurations, indicating that the comprehensiveness of the proposed Mamba-MSTN framework can bring excellent performance. **Figure 7** visualizes the ablation experiments. Although some simplified models occasionally show slight advantages on individual dates or indicators, these improvements are negligible and lack statistical significance. In contrast, deleting any key components (MSTFE, Mamba or MHSA) usually undermines the model’s ability to perform effectively. This outcome indicates that these three modules have made significant contributions to system performance and confirm the effectiveness of the Mamba-MSTN framework.

**Table 15:** Ablation study comparison (July 12).

Models	Sample size							
	90	120	150	180	270	360	720	1440
Mamba-MSTN	**92.58**	**93.91**	**94.65**	**95.68**	96.96	**97.49**	**98.75**	99.13
W/O-MSTFE	90.95	93.89	93.34	95.03	96.35	96.88	98.66	99.05
W/O-Mamba	89.94	92.86	93.42	94.30	**97.13**	97.45	98.37	**99.34**
W/O-MHSA	89.73	89.88	92.86	92.16	94.90	96.68	98.36	98.82

Best results are bolded.

**Table 16:** Ablation study comparison (July 13).

Models	Sample size							
	90	120	150	180	270	360	720	1440
Mamba-MSTN	**92.46**	**92.73**	93.54	**96.11**	**96.35**	**97.59**	98.34	99.27
W/O-MSTFE	91.16	92.22	93.90	95.55	95.67	97.02	**98.37**	99.03
W/O-Mamba	91.49	91.96	94.98	95.08	96.31	97.39	98.04	**99.48**
W/O-MHSA	85.90	89.69	90.56	92.59	94.92	96.37	98.14	99.42

Best results are bolded.

**Table 17:** Ablation study comparison (July 21).

Models	Sample size							
	90	120	150	180	270	360	720	1440
Mamba-MSTN	**55.94**	**63.04**	**68.67**	**74.97**	**85.18**	**87.39**	**92.37**	**94.84**
W/O-MSTFE	47.49	52.85	65.12	70.91	78.21	81.19	89.29	93.44
W/O-Mamba	42.48	57.12	68.02	68.44	70.88	71.66	72.03	75.00
W/O-MHSA	25.31	34.10	46.52	58.61	75.90	81.96	87.40	92.43

Best results are bolded.

**Table 18:** Ablation study comparison (July 23).

Models	Sample size							
	90	120	150	180	270	360	720	1440
Mamba-MSTN	**51.96**	**57.31**	**61.96**	**68.70**	**75.24**	**79.63**	**88.50**	**92.88**
W/O-MSTFE	46.45	53.86	56.61	63.08	71.72	77.22	87.71	93.54
W/O-Mamba	48.06	53.86	60.41	62.58	69.61	73.84	86.56	91.45
W/O-MHSA	44.08	47.08	53.54	61.52	66.44	75.24	85.45	91.00

Best results are bolded.

To understand how each module contributes to Mamba-MSTN, we ran ablation experiments across multiple datasets. These included binary-class data from July 12 and 13 and multi-class data from July 21 and 23, with sample sizes ranging from 90 to 1440. By comparing the complete model with variants lacking the MSTFE, Mamba, or MHSA module, we reveal how each module’s importance varies with task complexity and data availability.

For the binary classification tasks on July 12 and 13, under the condition of a minimum sample size of 90, the removal of MHSA will lead to the most significant performance decline, with a decrease of up to 6.56% on the data on July 13. This shows that global dependence modeling is the most critical when data is scarce, which helps to associate long-distance timing clues. The removal of Mamba will also cause a significant decline, with a decrease of about 2.6% to 2.9% on the data on July 12, reflecting its role in adaptive noise filtering. The MSTFE module brings a stable but relatively small performance improvement. When the sample size increases to 1440, the performance gap between the variant models almost disappears, and the occasional small advantages of the ablation model are statistically negligible, which confirms that the three modules jointly ensure the robustness of the model.

For the multi-classified tasks on July 21 and 23, the importance of each module is more prominent as the complexity of the task increases. In 90 samples, removing MHSA will cause the performance on the data on July 21 to drop sharply from 55.94% to 25.31%, which shows that global contextual information is indispensable for distinguishing between multiple types of faults under extreme data scarcity. Removing Mamba will also significantly reduce the performance on the July 21 data to 42.48%, while the removal of MSTFE will drop relatively mildly. It is worth noting that when the sample size increased to 1440, the accuracy of the variant of Mamba removal on the data on July 21 was only 75.00%, which was nearly 20% behind the complete model; while the variant that removed MHSA was significantly reduced to 92.43%. This shows that as the amount of data increases, Mamba’s adaptive filtering ability becomes more and more important, allowing it to make effective use of rich timing information. The MSTFE module continues to provide a stable multi-scale feature foundation, but it lack can be partially compensated by the remaining modules.

In a word, the ablation experiment confirms the unique contribution of each module: MHSA is indispensable for complex tasks under low data volume; the role of Mamba becomes more and more critical as the data volume increases; MSTFE provides stable multi-scale characteristics. These conclusions verify the rationality of the design of the proposed framework and can provide guidance for its adaptation in different real scenarios.

## 4 Discussion

In this study, to ensure the stability and high efficiency of plant protection technology and provide basic support for large-scale implementation, we have proposed an advanced UAVs fault diagnosis method called Mamba-MSTN. The framework integrates a MSTFE module, the Mamba-based adaptive state perception and screening module, and a global dependence modeling module using multi-head self-attention. It focuses on getting over the computational and time dynamics modeling constraints of current approaches. The suggested model has significantly improved overall performance and exhibits excellent application potential, according to the experimental results on the FW-UAVs data set. Specifically, in the binary fault diagnosis task, the suggested approach consistently outperforms the most advanced model available today in evaluating classification accuracy. This method’s accuracy rate is 1.92% greater than the current best model when 180 samples were used. When the sample size is 180 or more, the model maintains stable and robust performance in multiple classification tasks. When the sample size reaches more than 360, the accuracy rate is significantly improved, which obviously exceeds that of the comparison model. Besides, the Mamba-MSTN framework achieves high efficiency in computing processing and memory utilization.

Two key factors were identified by carefully examining the causes of reduced diagnostic accuracy in multi-class tasks under extremely small sample conditions. On the one hand, due to the strong feature correlations among different fault types in multi-class fault diagnosis, the model is prone to confusing decision boundaries between similar faults when trained with limited samples. On the other hand, the lack of adaptive initialization strategies specifically designed for low-sample scenarios during training prevents the model from effectively converging to an optimal state when data are insufficient. In contrast, multi-task learning methods such as HDMTL leverage feature-sharing mechanisms and regularization constraints, which provide stronger stability and robustness in low-sample settings. Future improvements may involve incorporating spatial feature analysis, self-supervised pretraining, meta-learning strategies, or integrating low-sample feature enhancement and class-adaptive weight distribution mechanisms to enhance efficiency of multi-type fault diagnosis in low-data scenarios.

Although SVM has achieved the best results in parameter counting and inference time, compared to proposed method, its diagnostic accuracy is substantially lower. Therefore, in general, the proposed method has successfully achieved the synergistic balance of high speed and diagnostic accuracy. This is mainly due to: The linear sequence modeling ability of the Mamba module, which avoids the attention mechanism’s secondary complexity. MSTFE module’s parallel design, combined with BiGRU and one-dimensional residual convolution blocks, improves the efficiency of feature extraction. The whole end-to-end joint training of individual architectures realizes the collaborative optimization of all components and reduces redundant computations.

While the suggested model already outperforms the majority of current approaches, its performance can be further enhanced in low-sample scenarios involving multiple types of fault diagnosis. However, there is little investigation of the spatial structural relationships between sensors in the current model, which primarily concentrates on temporal modeling. Future research will look in a number of ways to address this issue: In order to facilitate deeper joint modeling of spatial and temporal features, our first goal is to integrate graph-based structural modeling by looking into architectures like Graph Mamba. Second, we plan to incorporate self-supervised pre-training or meta-learning strategies, to leverage unlabeled data or task priors and enhance low-sample performance, thereby improving generalization and stabilizing model convergence. In addition, we will explore the deployment of a lightweight and interpretable fault diagnosis model on UAVs to integrates the applications of UAVs with practical plant protection requirements. This model aims to enhance trust in intelligent diagnostics and facilitate rapid responses to critical faults.

## Data Availability

Publicly available datasets were analyzed in this study. The raw data used in this paper can be obtained from Murat et al., 2020.
